# Advanced Platelet-Rich Fibrin Plus (A-PRF+) as an Additive to Hard Tissue Managing Protocols in Oral Surgery: A Systematic Review

**DOI:** 10.3390/jfb16040145

**Published:** 2025-04-19

**Authors:** Marek Chmielewski, Andrea Pilloni, Paulina Adamska

**Affiliations:** 1 Private Dental Practice, 14 Kolberga Street, 81-881 Sopot, Poland; 2 Section of Periodontics, Department of Oral and Maxillo-Facial Sciences, Sapienza Unviersity of Rome, 00-185 Rome, Italy; andrea.pilloni@uniroma1.it; 3 Department of Oral Surgery, Medical University of Gdańsk, 7 Dębinki Street, 80-211 Gdańsk, Poland

**Keywords:** advanced platelet-rich fibrin, A-PRF, autografts, dentistry, growth factors, wound healing

## Abstract

Background: Advanced platelet-rich fibrin + (A-PRF+) represents a third generation of autologous platelet derivatives. Appropriate centrifugation conditions cause the formation of a clot containing platelets, which slowly release growth factors that influence healing. The objective of this article was to undertake a review of the available literature on the effectiveness of A-PRF+ use in hard tissue procedures. Materials and methods: In order to ensure the most accurate and relevant results, only randomized clinical trials regarding bone regeneration techniques/bone healing that compared the effect of the A-PRF+ addition in dentistry were included in this study. Articles taken into consideration for the review were published between the beginning of 2014 and 31 December 2024. The search of manuscripts for the review was conducted using the PubMed, Scopus, Google Scholar, and Cochrane databases. For this study, 10 articles focusing on A-PRF+ were qualified. Results: A-PRF+ was found to increase the post-surgical vertical and horizontal alveolar ridge dimensions. The bone formed in the surgical site presented a higher volume of vital and non-vital bone and a more optimal bone composition, at the same time providing a lower percentage of connective tissue inclusions. When combined with other grafting biomaterials, A-PRF+ enhanced their performance and integration. A-PRF+ did not have any significant effect on the mineral bone density compared with other grafting materials. Compared with PRF and other blood derived plasmas rich in growth factors, the performance of A-PRF+ was generally better, but often with no statistical significance. The treatment of periodontal defects measured by the reduction in pocket depth and clinical attachment level also fared better with the A-PRF+ addition, although there was no differences noted between A-PRF+ and biphasic calcium phosphate and xenograft. Finally, the A-PRF+ addition improved the primary implant stability in the evaluated studies. Conclusions: The A-PRF+ addition to the surgical protocols significantly enhanced the healing of the bone and when combined with biomaterials improved their integration and increased the implant insertion torque, improving the primary and secondary stability. It may be a viable alternative for patients that express their concern towards human- and animal-derived biomaterials.

## 1. Introduction

Dr. Jospeh Choukroun is widely considered as a promoter of the use of blood-derived platelet-rich biomaterials rich in growth factors. Choukroun, together with his team, developed and distributed the first protocols for preparation at the beginning of the 21st century. Studies on blood-derived, platelet-rich preparations enriched with growth factors have been conducted to improve their biological and physical properties. Researchers aimed to develop preparations with greater cohesion, allowing for better positioning, particularly in bone defects during procedures in oral surgery, periodontology, and implantology, for example, after tooth extraction and in bone regeneration (bone augmentation, sinus lift procedures, treatment periodontitis, or during implant placement). Additionally, it was found that the new generations of these preparations contain novel growth factors and cytokines, which enhance their biological properties. This leads to reduced post-procedure reactions (such as pain and swelling), faster wound healing, and other benefits [1,2,3,4].

Available studies on blood-derived, platelet-rich preparations enriched with growth factors emphasize their role in enhancing healing and reducing procedural discomfort, particularly during the first days or up to two weeks after the procedure. This applies to research on platelet-rich plasma as well as various types of platelet-rich fibrins. This period is crucial for patient recovery, and minimizing discomfort and potential complications significantly improves the patient comfort and quality of life post-procedure. Additionally, combining these preparations with bone-derived and bone substitute materials has been shown to enhance healing outcomes, such as by increasing the effectiveness of bone augmentation, sinus lift procedures, and implant treatments. Therefore, incorporating them into treatment protocols for oral procedures may be a rational approach [4,5,6,7,8,9,10,11,12,13,14,15,16].

The advanced platelet-rich fibrin (A-PRF) manufacturing method was first introduced and described by Ghanaati et al. [5] and Choukroun [6] in 2014, which was further refined by Kobayashi to create A-PRF+ [7]. A-PRF is a biocompatible material obtained from the patient’s venous blood. The standard procedure for the production of A-PRF+ entails the collection of the peripheral venous blood of the patient and then the utilization of vertical centrifuging at a rotational speed of 1300–1500 rpm for a period of 14 min, as outlined by Kobayashi. A further variant, designated A-PRF+, has been developed, which requires a different centrifuging protocol of 1300 rpm for 8 min and was designed to further enhance the standard A-PRF protocol [7]. The vertical centrifugation process facilitates the separation and sedimentation of cellular components according to their respective molecular masses, as proposed by Herrera-Vizcaíno [8]. Moreover, a reduction in the centrifuge speed compared to the standard PRF procedure, which involves speeds between 2700–3000 rpm for a duration of 12 min, allows for a superior release of growth factors. It has been demonstrated that platelets remain present in the peripheries of the A-PRF+ clot. The discrepancy in processing may be accountable for the enhanced optimization, longevity, and more uniform distribution and liberation of growth factors from A-PRF into adjacent tissues, thereby influencing the regeneration and maturation of these tissues. The lymphocytes, macrophages, and stem cells are concentrated in the proximal part of the clot, whereas neutrophils are located mainly in the distal part [5,6,8]. A-PRF and A-PRF+ have been proven to contain cytokines and growth factors such as the following: bone morphogenetic proteins, fibroblast growth factor, matrix metalloproteinases, platelet-derived growth factor, transforming growth factors α and β, and vascular endothelial growth factor [16,17,18,19,20]. Due to the modified centrifugation parameters, A-PRF+ contains a higher concentration of growth factors and cytokines compared to A-PRF. This enhances its angiogenic properties and may improve tissue regeneration during healing. Additionally, its higher density increases the stability and retention in the wound compared to A-PRF. The A-PRF+ mesh traps more growth factors and neutrophils. This ensures good cohesion and attachment to the wound. The available evidence suggests that A-PRF (and its more recent iteration, A-PRF+) can release cytokines for up to 10 days. A-PRF+ is an additional tool that can be used to improve the result of the standard surgery procedure [6,9,10,11,12].

The objective of this article was to undertake a review of the available literature on the effectiveness of A-PRF+ use in hard tissue management procedures. This article provides a clear and structured summary of the available publications on the improved A-PRF formula and its role in oral surgery, periodontology, and implantology.

## 2. Materials and Methods

The review was carried out beginning with the search of the literature, which was conducted using PubMed, Scopus, Google Scholar, and Cochrane web databases to answer the questions: ‘Does A-PRF+ addition improve integration of bone grafting materials in oral surgery procedures?’ and ‘Does A-PRF+ addition improve hard tissue healing in oral surgery procedures?’ To search for relevant publications, the following MeSH terms were used: ‘platelet-rich fibrin’, ‘PRF’, ‘autografts’, ‘dentistry’, ‘growth factors’, and ‘wound healing’. The search was conducted for manuscripts published between 1 January 2014 and 31 December 2024.

This project was registered in the International Prospective Register of Systematic Reviews (PROSPERO) and granted the number CRD42023449648.

### 2.1. Inclusion and Exclusion Criteria

In order to ensure the most accurate and relevant results, only randomized clinical trials regarding bone regeneration techniques that compared the effect of A-PRF+ addition were included in this study. The studies were selected from the databases according to the following inclusion criteria: (1) only studies conducted on human subjects were included, (2) studies that employed A-PRF+ in conjunction with biomaterials for bone regeneration techniques, (3) studies that were carried out and published between the 1 January 2014 and 31 December 2024, (4) studies that were published in English, and (5) randomized clinical studies that included a minimum of 10 patients.

The exclusion criteria included the following: (1) studies not conducted on human subjects, (2) studies that employed A-PRF (not A-PRF+) in conjunction with biomaterials for bone regeneration techniques, (3) studies that employed A-PRF+ as an additive in managing soft tissue surgical protocols, (4) studies that were carried out and published before 2014 (before the development of A-PRF+), (5) studies that were not published in English, and (6) case reports and articles that included a less than 10 patients.

### 2.2. Screening and Data Extraction

Two reviewers (MC and PA) independently performed the data extraction. Any disagreements during the extraction process were resolved by discussion between the reviewers. Duplicates from databases were excluded. Lastly, the full-text manuscripts were subjected to a review in accordance with the established selection criteria. A total of 10 publications were deemed eligible for inclusion in this review.

## 3. Results

**Figure 1:**
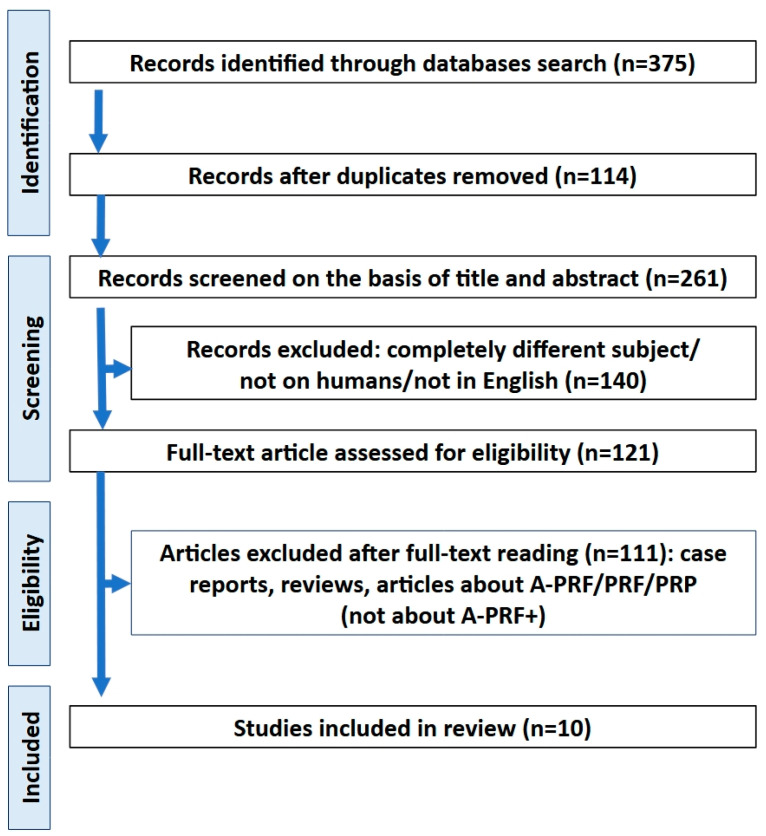
PRISMA flowchart.

In the initial stages of the selection process, a total of 217 references were identified through searches of the PubMed, Scopus, Google Scholar, and Cochrane databases. Following the removal of duplicates, 114 articles were retained. Following the screening of the titles and abstracts, 140 positions were excluded. Full texts were then read, and only publications that were randomized trials about A-PRF+ were included. Case reports, reviews, or articles about A-PRF/PRF/PRP (not specifically about A-PRF+) were excluded. Ultimately, 10 articles were deemed suitable for inclusion in this systematic review [13,14,15,16,17,18,19,20,21,22]. Subsequently, the remaining studies were divided into the appropriate categories according to the procedures they employed. The initial studies included in the review were published in 2015 (Figure 1 and Table 1).

### 3.1. A-PRF+ Effect on Vertical and Horizontal Alveolar Ridge Dimensions

A total of five randomized clinical trials were conducted to evaluate the alveolar ridge dimensions following tooth extractions and grafting procedures. In four out of the five studies, the incorporation of A-PRF+ into the surgical protocol resulted in an improvement in the clinical outcome or facilitated the use of co-used biomaterials, thereby producing more favorable results [13,14,15,16,17].

In the study conducted by Clark et al. [13], the blood clot was employed as a control. The test groups employed a freeze-dried bone allograft (FDBA), A-PRF+, or a combination of the two materials. Each test group demonstrated a superior performance in maintaining three-dimensional bone measurements in comparison to the control group. When employed independently, A-PRF+ demonstrated comparable outcomes to the FDBA with respect to the vertical and horizontal bone loss. The incorporation of A-PRF+ into the FDBA resulted in a notable enhancement in the vertical measurements. The incorporation of biomaterials did not exert a notable impact on the horizontal dimensions in comparison to the control group at the apical region of the alveolar ridge. However, the test group, comprising the FDBA and A-PRF+, demonstrated a significantly superior capacity to impede the loss of the coronal horizontal dimensions in comparison to all other groups.

A comparable trial was conducted by Ivanova et al. [14], in which the test group that had used the mixture of FDBA and A-PRF+ was omitted. In the study, A-PRF+ demonstrated a comparable performance to the FDBA. The results for both the FDBA and A-PRF+ groups in terms of the vertical and horizontal dimensions were statistically significant when compared to those of the blood clot control group. Both the Clark and Ivanova studies had approximately the same follow-up time of four months. The follow-up time in the Clark study was 3.75 months, while in the Ivanova study it was 4 months.

In their respective studies, Alhaj et al. [15] and Yewale et al. [16] employed A-PRF+ as an adjunct to the bone biomaterial. A minimal discrepancy between the groups was observed on the second day of the study by Alhaj et al. [15]. At the six-month mark, the control and test groups exhibited statistically significant differences in favor of the test group. Furthermore, Yewale et al. [16] observed that the addition of A-PRF+ enhanced the performance of other hard tissue grafting materials. The most significant gains were observed in the horizontal dimensions, particularly at the 3 mm and 5 mm measuring points.

Kalash et al. [17] employed an xenograft material with (test) and without (control) A-PRF+. The results of this study differ from those of the others, as the resorption margin in the test group was found to be higher than in the control group. No statistically significant difference was observed between the two groups. The results are presented in Table 2 [13,14,15,16,17].

### 3.2. A-PRF+’s Effect on Vital Bone Formation, Grafting Material Turnover, and Percentage of Connective Tissue in Grafting Site

The grafting material integration, the turnover, and the formation of bone and connective tissue were investigated in five randomized controlled trials. The incorporation of A-PRF+ into the protocol resulted in an enhanced tissue formation when compared with the control groups and facilitated a more effective redistribution of the grafting material at the surgical sites [13,14,18,19,20].

All of the studies considered reported on the formation of the bone tissue. In the study conducted by Clark et al. [13], the incorporation of a single biomaterial resulted in a reduction in the vital bone formation when compared to the other experimental groups (29% vs. 40–46%). However, when the same biomaterial was combined with A-PRF+, the percentage of vital bone increased to a level comparable to that observed in the control group. Furthermore, the incorporation of A-PRF+ resulted in a notable reduction in the connective tissue percentage; however, in the Clark et al. study, this decline was relatively minor. Similarly, Ivanova observed a favorable impact of the A-PRF+ supplementation on the bone vitality in comparison to the control group. No statistically significant difference was observed between the two test groups. Furthermore, the addition of A-PRF+ has been observed to significantly influence the quantity of the connective tissue present within the harvested specimen. The addition of A-PRF+ had a comparable effect to the FDBA, which inhibited the formation of connective tissue and promoted bone regeneration [13]. Additionally, Hartlev et al. [18] investigated the quantity of non-vital bone. A comparison of the A-PRF+ and deproteinized bovine bone mineral (DBBM) groups revealed comparable levels of vital bone. The two groups exhibited notable differences in the non-vital bone percentage (A-PRF+ 80%, DBBM 63%) and the percentage of connective tissue (5% vs. 22%). In comparison to the DBBM, the A-PRF+ specimens exhibited a greater proportion of non-vital bone, accompanied by a reduction in the connective tissue within the bone structure. Dragonas et al. [19] and Lavagen et al. [20] also employed the DBBM grafting material in their study, yet no differentiation was made between vital and non-vital bone. The addition of A-PRF+ in Dragonas et al. [19]’s study resulted in a greater extent of bone formation than that observed in the sole DBBM control group (20.33 ± 11.5% vs. 32.2 ± 7.29%), although this was similar to what was observed in another test group that used plasma rich in growth factors (PRGF) (32.2 ± 7.29% vs. 34.8 ± 6.83%). The increase in the bone formation observed in both test groups in comparison to the control group was statistically significant. The turnover of the material in the A-PRF+ group was slower than in the control group (24 ± 7.94% vs. 26 ± 7.78%) and in the PRGF group (24 ± 7.94% vs. 15.8 ± 8.23%). The percentage of connective tissue in the A-PRF+ group was found to be lower than that of the control group (41.4 ± 8.32% vs. 55.66 ± 7.77%) and the PRGF group (41.4 ± 8.32% vs. 49.6 ± 5.68%). The study conducted by Lavagen et al. [20] concentrated exclusively on the percentage of newly formed bone. In contrast with the findings of other studies, both the test and control groups employed the use of a blood clot derivative. The control group employed an older generation of PRF, whereas the test group utilized A-PRF+. The test group exhibited superior outcomes compared to the control group, with a statistically significant difference in the formation of new bone, reaching 60.4 ± 10.4% compared to 51.4 ± 18.4% for the control group. The mean volume of the newly formed bone for the test group was 0.29 ± 0.09 cm^3^, while that for the control group was 0.2 ± 0.08 cm^3^, indicating that the gain in the A-PRF+ group was statistically significant.

In the study by Ivanova et al. [14], there was no statistical difference between the allograft and A-PRF+ for the vital bone formation. The results were statistically better compared to the control group. The same observations were made in the case of the connective tissue graft. The results are presented in Table 3 [13,14,18,19,20].

### 3.3. A-PRF+’s Effect on Obtained Bone Mineral Density

Four randomized controlled trials that focused on bone density were identified. The incorporation of A-PRF+ into the existing protocols did not yield statistically significant improvements between the test and control groups [13,15,16,17].

Clark et al. [13] conducted a measurement of bone density using specimens obtained during implant placement surgeries and presented the results in mg/cm^3^. The addition of A-PRF+ to the FDBA did not result in an improvement in mineral bone density; in fact, it led to a decrease in this parameter (521 ± 58 mg/cm^3^ vs. 551 ± 58 mg/cm^3^). However, the incorporation of any biomaterial, whether A-PRF+ or FDBA, demonstrated superior outcomes compared to the control group (487 ± 64 mg/cm^3^).

Alhaj et al. [15] conducted a voxel count analysis on superimposed X-ray images at the six-month follow-up. The difference between the control group (802.5 voxels) and the test group (827.5 voxels) was not statistically significant. However, the addition of A-PRF+ to the autologous bone graft resulted in an increase in the clinical performance (246.5 with A-PRF+ vs. 167.25 without A-PRF+, mean difference between baseline and 6-month follow-up).

Two of the studies [16,17] employed a comparison of bone density in Hounsfield Units (HUs) at 6-month [16] and 9-month [17] follow-ups. The study by Kalash et al. [17] did not yield statistically significant results at the 9-month control point. The addition of A-PRF+ to the biomaterial (Xenograft) resulted in only a slight improvement in the mean result (496.86 ± 43.98 HU without A-PRF+ compared with 518.14 ± 45.24 HU with A-PRF+). Furthermore, Yewale et al. [16] did not present a statistically significant difference, with a total mean difference between the baseline and the 6-month follow-up (1393.1 HU control vs. 1783.1 HU test group). However, the bone density with the addition of A-PRF+ was higher. The results are presented in Table 4. The authors corrected to adhere to the one unit of measurement (HU as most of the X-ray programs enable comparison in these units). Thanks to this, the studies can be compared. But, the limitation is that the grey values, HU and histological mg HA cm^−3^, are not inherently interchangeable. Converting either HU or CBCT grey values to mg cm^−3^ requires a scanner-specific hydroxyapatite phantom calibration, which none of the included studies reported [13,15,16,17].

### 3.4. A-PRF+’s Effect on Pocket Depth (PD) and Clinical Attachment Level (CAL)

Two randomized controlled trials considered the alterations in the pocket depths (PDs) and clinical attachment levels (CALs) subsequent to surgical procedures [17,21].

In their study, Ghonima et al. [21] employed biphasic calcium phosphate (BCP), both with and without the addition of A-PRF+. The changes in the PD and CAL were monitored at three-month intervals, up until the ninth month of the final follow-up period. In comparison to the control group, both the BCP and the addition of A-PRF+ demonstrated significantly superior outcomes. At the initial two check-ups, the incorporation of A-PRF+ resulted in a reduction in PD levels, with no notable differences between the BCP and BCP + A-PRF+ groups (1.95 ± 0.9 mm vs. 1.5 ± 0.74 mm at three months, 2.18 ± 1.27 mm vs. 1.95 ± 0.56 mm). At the final follow-up, the PD in the BCP group was observed to be lower than that observed in the A-PRF+ group (2.04 ± 0.96 vs. 2.27 ± 0.71 mm), though the difference in the results between the two groups was not statistically significant. Similarly, the initial CALs remained lower in the group that received A-PRF+ (1.63 ± 0.89 vs. 1.36 ± 1.16 mm), before becoming higher than the sole BCP group at the 6- and 9-month controls (1.68 ± 1.23 vs. 1.77 ± 0.68 mm and 1.68 ± 1.23 vs. 2.13 ± 1.02 mm, respectively).

Kalash et al. [17] only evaluated the PD changes at the 3- and 6-month controls, without assessing the CAL. The addition of A-PRF+ to the protocol resulted in a decrease in the average PD at both the three- and six-month follow-ups (3.39 ± 0.4 vs. 2.96 ± 0.64 mm and 2.89 ± 0.28 vs. 2.57 ± 0.51 mm, respectively). The statistical analysis revealed that the differences between the xenograft and the xenograft + A-PRF+ remained insignificant. The results are presented in Table 5 [17,21].

### 3.5. A-PRF+’s Effect on Obtained Implant Stability

Two randomized controlled trials, conducted by Kalash and Angelo, were identified that measured either the primary or secondary implant stability [17,22].

Kalash et al. employed the PerioTest (PTV—PerioTest value; physiological mobility PTV −08 to +09; I grade of mobility +10 to +19; II grade of mobility +20 to +29; III grade of mobility +30 to +50) device to assess the secondary stability at the three- and six-month follow-up periods. The group that utilized A-PRF+ demonstrated superior outcomes in both measurement intervals when compared to the group that solely employed xenograft. At the three-month mark, the xenograft cohort attained a score of −4.14 ± 1.06, while the combination of xenograft and A-PRF+ reached a score of −5.47 ± 1.16. At the final follow-up, six months after surgery, the PerioTest score for the xenograft group exhibited further improvement, reaching −4.51 ± 0.94, while the xenograft + A-PRF+ score demonstrated a similar trend, reaching −6.14 ± 1.27. The differences in the PerioTest score between the groups were statistically significant [17].

Angelo et al. [22] conducted an assessment of the primary stability during the implant insertion procedure. A comparison was made between the native bone and two variants of the EasyGraft product, namely B-TCP Crystal and Classic. Additionally, the Classic variant was augmented with A-PRF+, thereby facilitating a comparison between the sole EasyGraft Classic. The lowest mean torque values for implant insertion were observed in the native bone (control) group (27.87 ± 6.66 Ncm). All biomaterials demonstrated significantly higher average results, with the highest observed in the EasyGraft Crystal group (52.5 ± 8.15 Ncm). The difference between the EasyGraft Classic (42.51 ± 7.03 Ncm) and Crystal (52.5 ± 8.15 Ncm) variants was found to favor the latter. The incorporation of A-PRF+ into the EasyGraft Classic resulted in an enhanced primary implant stability (42.51 ± 7.03 Ncm vs. 46.89 ± 4.57 Ncm). The results are presented in Table 6.

## 4. Discussion

The primary objective of this systematic review was to evaluate the effectiveness of A-PRF+’s addition to surgical protocols aimed at managing hard tissues in the oral cavity, both as a sole material and as an additive to grafting materials used for bone augmentation. The present study was conducted based on the premise of randomized clinical trials, which, given their meticulous methodology, yield results that present the highest clinical value. However, it should be noted that such trials can impose certain limitations [23]. One such limitation that was present in our study was the size of the study group of participants, with the lowest patient count being 15 in total. Only two studies presented large patient study groups of over 60 patients. However, it is noteworthy that other studies have amassed sufficient patient counts, thereby mitigating potential biases associated with inadequate participant numbers (328 patients in total across ten studies incorporated in this systematic review). The collective evidence was appraised to be of a moderate to high quality. A notable limitation pertains to the utilization of diverse protocols, each tailored to address the unique requirements of the patient undergoing the respective procedures. Despite the maintenance of the general protocol of A-PRF+ creation, as suggested by Fujioka-Kobayashi et al. [7], there were slight variations in the intraoperative and post-operative methods among the studies. As in daily practice, every surgeon follows a protocol that has been adapted to suit the operator’s needs in order to achieve the best possible outcome. This discrepancy complicates the direct comparison between the studies, underscoring the necessity for further research in this area to establish clear guidelines. The post-operative care regimen differed most often during the early healing process, with subsequent follow-ups and clinical/radiological evaluations scheduled at 3 and 6 months.

In the studies presented, A-PRF+ was most frequently utilized as a singular biomaterial in the post-extraction socket, with the objective of preserving its three-dimensional characteristics. The results obtained from the patients’ follow-up clearly demonstrated the superiority of the A-PRF+ addition into the protocol. When compared with other blood-derived platelet-rich fibrins, A-PRF+ did not yield any statistically significant results. However, it is noteworthy that A-PRF and A-PRF+ exhibit the highest growth cytokine concentrations among all blood derivatives. Consequently, a comparison with L-PRF can only be made in terms of clinical outcomes in bone management [24]. The increase in the growth cytokines concentration is attributable to the increased neutrophil levels trapped in the fibrin scaffolding during a slower and shorter centrifugal process [5], with A-PRF+ demonstrating superiority over conventional A-PRF. The increased growth cytokines concentration translates to a higher and faster response for the neoangiogenesis and mRNA response, with VEGF highlighted as a main factor for this response [25,26].

In many cases, surgical protocols incorporated the utilization of A-PRF+ in conjunction with bone grafting materials. The rationale for the incorporation of A-PRF+ in bone grafting materials is consistent with the rationale for its sole use, i.e., to maintain the dimensions of the alveolar ridge at the extraction site. The rationale for this choice is based on the density and availability of growth factors provided by the fibrin, which promote the angiogenesis process and mobilize the cellular response to utilize the grafting material as efficiently as possible [27]. The protocol employed by clinicians has been found to influence the grafting material’s turnover rate and the subsequent formation of vital and non-vital bone. While the studies have demonstrated a general trend in favor of A-PRF+ utilization, further research is necessary to ascertain its optimal application. In a similar vein to studies solely employing A-PRF+, no statistically significant difference was observed between other blood-derived biomaterials.

The most contentious issue that emerged from the collective analysis of the studies was the assessment of the benefits associated with the collection of patient blood during surgical procedures. This is the one common concern for all of the blood derivatives. In particularly complex cases, heightened scrutiny was applied, given the A-PRF+ preparation’s necessity for additional procedures (e.g., blood drawing, centrifugation, and blood clot separation), instruments (e.g., centrifuge machine, glass/plastic tubes, and PRF box), and expenses. As is often the case in clinical practice, the decision regarding the use of A-PRF+ must be made in consultation with the patient, after a thorough discussion of the potential benefits and drawbacks of its implementation in the specific clinical context. As surgical procedures become increasingly intricate, concerns regarding the utilization of A-PRF+ may diminish, as its benefits for bone and grafting material management are indisputable. In such cases, the financial implications, when considered in relation to the overall treatment costs, become a relatively less significant factor.

During the preparation of this systematic review, the primary concern for future studies was the focus on larger study groups and the use of various grafting materials in surgical protocols to better evaluate their effectiveness in oral surgery and implantology. In this study, the bone grafting materials exhibited substantial heterogeneity, as xenografts, allografts, and alloplastic materials were utilized. The paucity of studies conducted on limited study group populations engenders a considerable difficulty in comparing the effects of A-PRF+’s addition to different bone grafts and bone substitute materials. The wide range of clinical protocols created by clinicians to best suit their needs and the various methods of testing the effectiveness of the procedure (CBCT measurements, bone density measurements using grey point or Hounsfield Units, bone sample collections and their immunohistographic evaluation) create another layer of comparative difficulty for the results. For procedures necessitating flap reopening and bone access, the acquisition and subsequent immunohistographic evaluation of bone samples would provide the most bias-proof evidence regarding the clinical outcome, grafting material incorporation and turnover, and bone behavior. For the least invasive approach, it would be valuable to assess the therapeutic success using Hounsfield Units on CBCT and CT and compare their quality, as the procedure of choice to evaluate any three-dimensional changes at the follow-up visit is taking the CBCT or CT.

## 5. Conclusions

In summary, the analyzed studies suggest that A-PRF+ provides a superior stabilization of ridge dimensions following tooth extraction compared to a natural blood clot. Furthermore, when combined with xenografts or alloplastic materials, A-PRF+ may enhance periodontal tissue regeneration, facilitate bone augmentation, and contribute to improved implant stability.

## Figures and Tables

**Table 1:** Studies included in review.

No.	References	Country	Number of Patients	Preparation of A-PRF
1	Alhaj et al., 2018 [15]	Lebanon	20	1300 rpm for 8 min (A-PRF+)
2	Angelo et al., 2015 [22]	Austria	82	A-PRF+ (exact preparation not specified)
3	Clark et al., 2018 [13]	USA	40 (divided into 4 groups)	1300 rpm for 8 min (A-PRF+)
4	Dragonas et al., 2023 [19]	USA	15	1300 rpm for 5 and 3 min (8 min total) with liquid layer removal after 5 min centrifuge (A-PRF+ modified protocol)
5	Ghonima et al., 2020 [21]	Egypt	22	1300 rpm for 8 min (A-PRF+)
6	Hartlev et al., 2020 [18]	Denmark	27 (divided into 2 groups)	1300 rpm for 14 min (A-PRF+ modified)
7	Ivanova et al., 2019 [14]	Bulgaria	60 (divided into 3 groups)	1300 rpm for 8 min (A-PRF+)
8	Kalash et al., 2017 [17]	Lebanon	18	1300 rpm for 8 min (A-PRF+)
9	Lavagen et al., 2021 [20]	France	24	1300 rpm for 5 and 3 min (8 min total) with liquid layer removal after 5 min centrifuge (A-PRF+ modified protocol)
10	Yewale et al., 2021 [16]	India	20	1300 rpm for 8 min (A-PRF+)

**Table 2:** Differences in vertical and horizontal dimensions compared to control and other materials.

No.	References	Vertical Dimensions Loss (mm)						Horizontal Dimensions (mm)					
1	Alhaj et al. [15]	Marginal bone height		Autologous bone graft	Autologous bone graft + A-PRF+		*p* value	No data					
		Day 2		0.515	0.435		0.009						
		6 months		0.205	0.35		<0.001						
2	Clark et al. [13]	15 weeks	Blood clot	A-PRF+	FDBA + A-PRF+ (1:1 ratio)	FDBA	*p* value	15 weeks	Blood clot	A-PRF+	FDBA + A-PRF+ (1:1 ratio)	FDBA	*p* value
			3.8 ± 2	1.8 ± 2.1	1.0 ± 2.3	2.2 ± 1.8	Blood clot vs. A-PRF+ and FDBA + A-PRF+ <0.05	Coronal	2.9 ± 1.7	2.8 ± 1.2	1.9 ± 1.1	2.5 ± 1.1	FDBA + A-PRF+ < 0.05 vs. other group
								Middle	1.8 ± 1.3	1.8 ± 1.8	1.7 ± 1.2	1.5 ± 1.2	
								Apical	1.6 ± 1.5	1.8 ± 1.5	1.6 ± 1.5	1.2 ± 1.3	
3	Ivanova et al. [14]		Blood clot	A-PRF+		FDBA	*p* value		Blood clot	A-PRF+		FDBA	*p* value
		4 months	1.38 ± 0.13	0.87 ± 0.21		0.91 ± 0.24	A-PRF+ and FDBA > 0.05; blood clot < 0.05	4 months	2.39 ± 0.4	1.52 ± 0.31		1.33 ± 0.25	A-PRF+ and FDBA > 0.05; blood clot < 0.05
4	Kalash et al. [17]	Marginal bone height		Xenograft	Xenograft + A-PRF		*p* value	No data					
		Baseline		1.79 ± 0.2	1.91 ± 0.22								
		9 months		1.3 ± 0.32	1.37 ± 0.2								
5	Yewale et al. [16]	6 months		Sybograf plus (70% HA, 30% BTCP) + A-PRF+:		Sybograf plus (70% HA, 30% BTCP	*p* value	6 months	Sybograf plus (70% HA, 30% BTCP) + A-PRF+		Sybograf plus (70% HA, 30% BTCP		*p* value
				1.4		1.67	<0.05	Mean difference	Socket width at 1 mm			
									2.12		1.83		0.49
									Socket width at 3 mm			
									1.68		0.59		0.041
									Socket width at 5 mm			
									0.97		0.33		0.65

A-PRF+—advanced platelet-rich fibrin +; BTCP—β-tricalcium phosphate; FDBA—freeze-dried bone allograft; and HA—hydroxyapatite.

**Table 3:** Differences in vital bone, non-vital bone, and connective tissue formation and residual grafting material (residual material does not apply to A-PRF+, only FDBA and DBBM).

No.	References	Vital Bone (%)					Non-Vital Bone (%)			Residual Material (%)					Connective Tissue (%)				
1	Clark et al. [13]	Blood clot	A-PRF+	A-PRF+ and FDBA (1:1 ratio)	FDBA	*p* value	No data			Blood clot	A-PRF+	A-PRF+ and FDBA (1:1 ratio)	FDBA	*p* value	Blood clot	A-PRF+	A-PRF+ and FDBA (1:1 ratio)	FDBA	*p* value
		40 ± 18	46 ± 18	40 ± 15	29 ± 14	<0.05 (for A-PRF group)				0	0	3 ± 3	11 ± 9	>0.05	60 ± 10	55 ± 15	58 ± 5	58 ± 10	>0.05
2	Dragonas et al. [19]	Control (DBBM)			Test (A-PRF+ + DBBM)		Test (PRGF + DBBM)		*p* value	Control (DBBM)		Test (A-PRF+ + DBBM)	Test (PRGF + DBBM)	*p* value	Control (DBBM)	Test (A-PRF+ + DBBM)	Test (PRGF + DBBM)		*p* value
		20.33 ± 11.5			32.2 ± 7.9		34.8 ± 6.83		0.0875	24 ± 7.94		26 ± 7.78	15.8 ± 8.23	0.161	55.66 ± 7.77	41.4 ± 8.32	49.6 ± 5.68		0.0573
3	Hartlev et al. [18]	A-PRF		DBBM		*p* value	A-PRF	DBBM	*p* value	No data					A-PRF		DBBM		*p* value
		13.75 ± 13.18		14.16 ± 10.11		1.00	80.06 ± 15.28	63.16 ± 29.81	0.19						4.89 ± 6.05		21.96 ± 21.96		0.11
4	Ivanova et al. [14]	Blood clot		A-PRF	FDBA	*p* value	No data			Blood clot		A-PRF	FDBA	*p* value	Blood clot	A-PRF	FDBA		*p* value
		36.9 ± 14.94		60.48 ± 9.88	65.92 ± 10.91	<0.05				9.36 ± 6.49		10.99 ± 6.39	9.59 ± 5.38	>0.05	53.7 ± 17.79	28.53 ± 8.66	24.37 ± 9.35		>0.05
5	Lavagen et al. [20]	Control (iliac bone graft + PRF)				Test (iliac bone graft + A-PRF)			*p* value	No data					No data				
		Percentage of newly formed bone																	
		51.4 ± 18.4				60.4 ± 10.4			0.165										
		Mean volume of newly formed bone (cm^3^)																	
		0.2 ± 0.08				0.29 ± 0.09			0.024										

A-PRF+—advanced platelet-rich fibrin +; DBBM—deproteinized bovine bone mineral; FDBA—freeze-dried bone allograft; and PRGF—plasma rich in growth factors.

**Table 4:** Differences in mineral bone density.

No	References	Bone Mineral Density				
1	Alhaj et al. [15]			Autologous bone graft	Autologous bone graft + A-PRF	*p* value
		Day 2		635.25 voxel (−388.72 HU)	581 voxel (1024 HU)	0.481
		6 months		802.5 voxel (−221.5 HU)	27.5 voxel (996.5 HU)	0.684
2	Clark et al. [13]	A-PRF+	A-PRF+ and FDBA (1:1 ratio)	FDBA	Blood clot	*p* value
		493 ± 70 mg/cm^3^ (655 HU)	521 ± 58 mg/cm^3^ (701.7 HU)	551 ± 58 mg/cm^3^ (751.7 HU)	487 ± 64 mg/cm^3^ (645 HU)	<0.05
3	Kalash et al. [17]			Xenograft	Xenograft + A-PRF	*p* value
		Baseline		465.71 ± 48.57 HU	471.86 ± 51.64 HU	0.823
		9 months		496.86 ± 43.98 HU	518.14 ± 45.24 HU	0.39
4	Yewale et al. [16]			Test group	Control group	*p* value
		6 months		1783.1 HU	1393.1 HU	0.005

A-PRF+—advanced platelet-rich fibrin +; FDBA—freeze-dried bone allograft; and HUs—Hounsfield Units (values in mg/cm^3^ are shown in brackets for uniformity).

**Table 5:** Effect of A-PRF+ addition on PD and CAL.

No	References	Pocket Depth (mm)				Clinical Attachment Level (mm)			
1	Ghonima et al. [21]		BCP	BCP + A-PRF	*p* value		BCP	BCP + A-PRF	*p* value
		3 months	1.95 ± 0.9	1.5 ± 0.74	0.93	3 months	1.63 ± 0.89	1.36 ± 1.16	0.63
		6 months	2.18 ± 1.27	1.95 ± 0.56	0.6	6 months	1.68 ± 1.23	1.77 ± 0.68	0.66
		9 months	2.04 ± 0.96	2.27 ± 0.71	0.06	9 months	1.68 ± 1.23	2.13 ± 1.02	0.06
2	Kalash et al. [17]		Xenograft	Xenograft + A-PRF	*p* value	No data			
		3 months	3.39 ± 0.4	2.96 ± 0.64	0.09				
		6 months	2.89 ± 0.28	2.57 ± 0.51	0.173				

A-PRF+—advanced platelet-rich fibrin +; BCP—biphasic calcium phosphate.

**Table 6:** Differences in implant stability and insertion torque.

No	References	Implant Stability and Insertion Torque					
1	Kalash et al. [17]	PerioTest (PTV)	Xenograft		Xenograft + A-PRF+		*p* value
		3 months	−4.14 ± 1.06		−5.47 ± 1.16		0.045
		6 months	−4.51 ± 0.94		−6.14 ± 1.27		0.018
2	Angelo et al. [22]	Implant insertion torque (Ncm)	Native bone	Easy Graft Crystal	Easy Graft Classic	Easy Graft Classic + A-PRF	*p* value
			27.87 ± 6.66	52.5 ± 8.15	42.51 ± 7.03	46.89 ± 4.57	<0.05

A-PRF+—advanced platelet-rich fibrin plus; PTV—PerioTest value.

## Data Availability

The data presented in this study are available on request from the corresponding author. The data are not publicly available due to privacy restrictions.

## Abbreviations

A-PRF	advanced platelet-rich fibrin
A-PRF +	advanced platelet-rich fibrin plus
BTCP	β-tricalcium phosphate
BCP	biphasic calcium phosphate
CAL	clinical attachment level
DBBM	demineralized bovine bone mineral
FDBA	freeze-dried bone allografts
HU	Hounsfield Unit
PD	pocket depth
PRF	platelet-rich fibrin
PRGF	plasma rich in growth factors
PROSPERO	Prospective Register of Systematic Reviews
